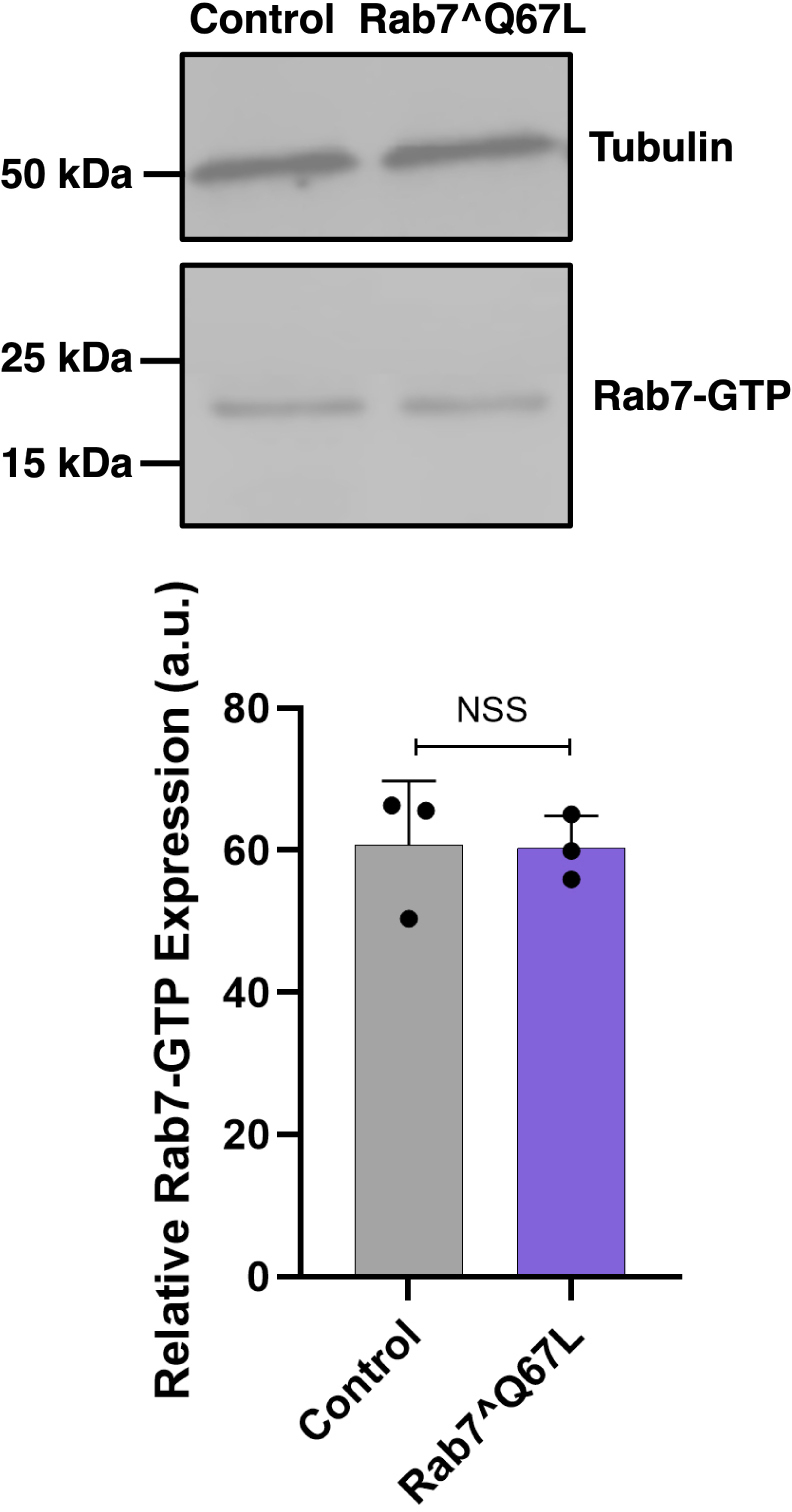# Sodium proton exchanger NHE9 pHine-tunes exosome production by impairing Rab7 activity

**DOI:** 10.1016/j.jbc.2025.108264

**Published:** 2025-02-03

**Authors:** Mariam Duhaini, Perla Fares, Lili Hafezi, Hadi El-Zein, Kalyan C. Kondapalli

**Affiliations:** Department of Natural Sciences, University of Michigan-Dearborn, Dearborn, Michigan, USA

**Keywords:** exocytosis, exosome, extracellular vesicle, NHE9, pH, SLC9A9, sodium-proton exchange

## Abstract

Cell-to-cell communication is mediated by vesicles ranging from 30 to 150 nm, known as exosomes. These exosomes shuttle bioactive molecules such as proteins, lipids, and nucleic acids, thus playing crucial roles in both health and disease mechanisms. Exosomes form within the endocytic pathway through the process of inward budding of the endosomal membrane, facilitated by the progressive acidification of the endosomal lumen. Although endosomal pH is known to be critical for exosome production, the precise molecular mechanisms involved remain poorly defined. Maintaining optimal endosomal pH involves meticulous coordination between proton pumping and leakage mechanisms. The sodium-proton exchanger NHE9, located on the endosomal membrane, modulates endosomal pH by transporting protons out of the endosomes in exchange for sodium or potassium ions. Here, we use genetic engineering, biochemistry, and advanced microscopy to demonstrate that the sodium-proton exchanger NHE9 significantly affects exosome production by regulating endosomal pH. NHE9-mediated endosomal alkalization impairs Rab7 activation, thereby disrupting the delivery of multivesicular endosomes to lysosomes. Moreover, luminal alkalization promotes the recruitment of Rab27b. This enhances the targeting of multivesicular endosomes to the cell periphery, their fusion with the plasma membrane, and subsequent exosome secretion. Our findings reveal the detailed molecular mechanisms through which endosomal pH regulates exosome production. Additionally, we identify NHE9 as a potential target for therapeutic strategies aimed at controlling exosome dynamics.

Exosomes are microscopic packets (30–150 nm) essential for cell-to-cell communication (1). They impact a diverse array of both physiological and pathological processes by facilitating the transfer of a complex molecular repertoire, including proteins, lipids, and nucleic acids. Their complex roles are closely tied to their intricate biogenesis process within the cell. During the maturation of endosomes, the endosomal membrane buds inward, selectively packaging specific proteins, lipids, and cytosolic constituents to form intraluminal vesicles (ILVs) (2). Enclosed within multivesicular endosomes (MVEs), these ILVs may either fuse with lysosomes for degradation or travel to the cell surface, where they are released as exosomes (2). Importantly, exosome secretion is not a random, ongoing process. Instead, it is a responsive mechanism triggered by cellular stimulation or occurring during certain pathological states (2). Despite their critical roles in biological processes, the precise mechanisms underlying exosome formation and secretion remain unclear. Among the various intracellular factors that regulate MVE trafficking, endosomal pH has emerged as an important yet unclear determinant (1).

Canonical exosome biogenesis begins at the plasma membrane, with endosome formation trapping extracellular fluid and establishing an initial luminal pH of approximately 7.2 to 7.4 (1). These endocytic vesicles gradually acidify as they mature (1). The pH transition plays a vital role in determining the fate of endosomal cargo, directing it toward lysosomal degradation, recycling to the plasma membrane, sorting through the trans-Golgi network, or packaging into MVEs for exosome release (3, 4, 5). Although the significance of luminal pH in exosome generation is recognized, the current understanding remains rudimentary and sometimes conflicting (6, 7, 8, 9, 10). Additionally, the common use of broad-acting agents such as ammonium chloride, chloroquine, and acidic media in past studies poses limitations, as these methods do not allow for the selective fine-tuning of endosomal pH required for precise investigations (6, 7, 8, 9, 10). Recently, induction of endolysosomal stress has been utilized to study exosome biogenesis, a process that incidentally alters luminal pH (11). However, this technique has drawbacks, as it could potentially impact normal physiological processes, which can complicate the interpretation of results. Thus, there is a pressing need for more selective approaches to study luminal pH's role in exosome biogenesis without disrupting normal cellular physiology.

The pH within endosomes is governed by the dynamic balance between V-ATPase–driven acidification and opposing forces such as proton leakage and counterion conductance (12). It is widely acknowledged that organellar pH is set not by direct regulation of V-ATPase activity but rather through pathways like proton-leak mechanisms, particularly involving Na^+^/H^+^ exchangers (NHEs), and is further fine-tuned by counterion channels, including Cl^−^ channels (13, 14). NHE isoform 9 (NHE9), localized selectively on endosomes is crucial in fine-tuning the pH within these compartments (15, 16, 17, 18). In this study, we show that a targeted elevation of pH within endosomes, specifically through the action of NHE9, leads to a significant increase in exosome production. An NHE9-mediated increase in late endosomal pH impairs Rab7 activation, thereby hindering the transport of MVEs to lysosomes. Furthermore, it promotes Rab27b recruitment, leading to increased fusion with the plasma membrane. This dissection of the molecular mechanisms fills a significant gap in our understanding of how endosomal pH can regulate exosome output and positions NHE9 as a promising target for modulating exosome yield for therapeutic applications.

## Results

### Exosome production varies with cellular NHE9 expression levels

**Figure 1:**
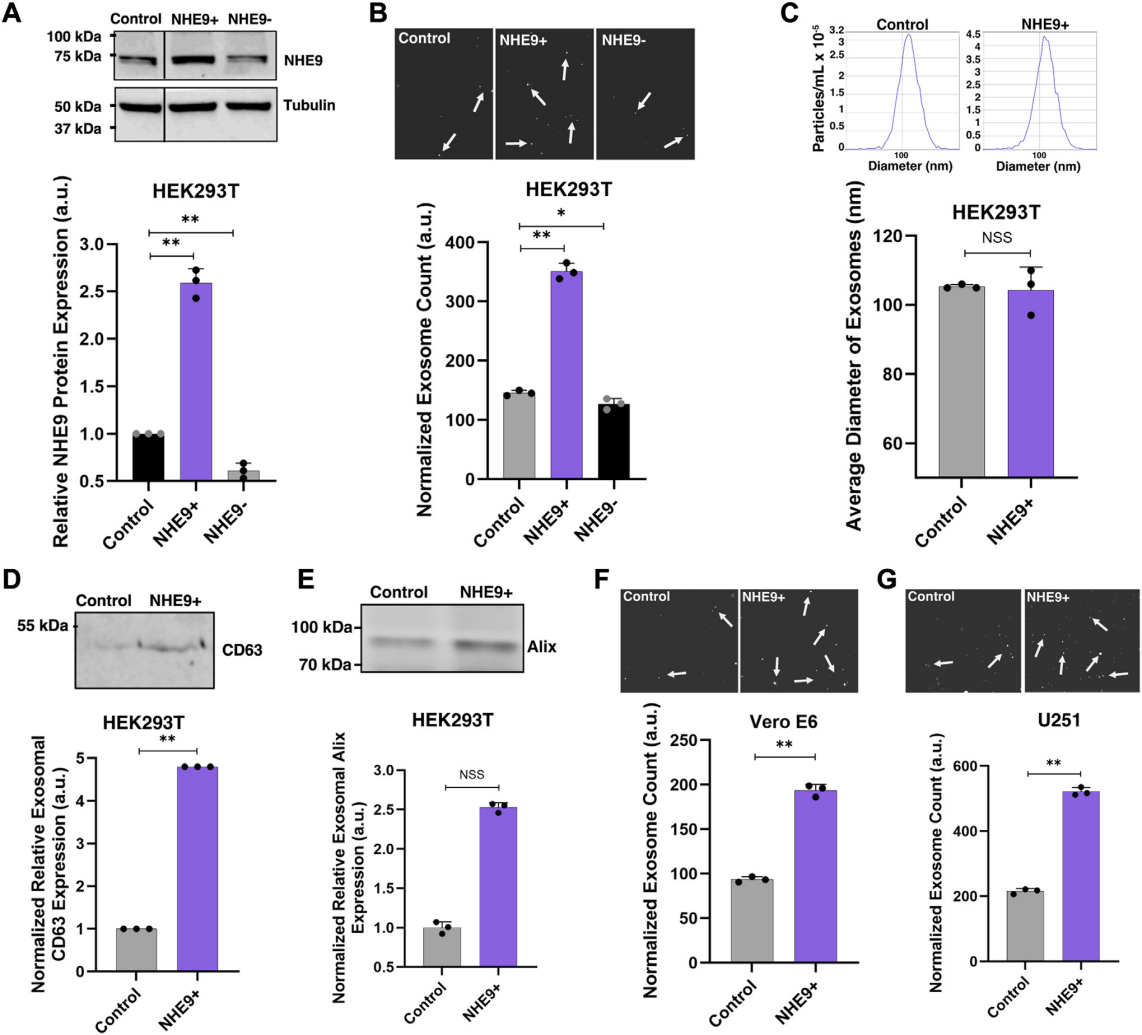
**NHE9 expression modulates exosome production across diverse cell lines.***A*, *upper panel*: representative immunoblot showing NHE9 expression in control HEK293T cells, NHE9-overexpressing (NHE9+), and NHE9 knockdown in NHE9-overexpressing cells (NHE9-). *Lower panel*: graphical representation of the average band intensity from densitometric scans of immunoblots from three biological replicates. Error bars indicate SD. ∗∗*p* < 0.005; statistical analysis performed using Student’s *t* test. *B*, *upper panel*: representative ZetaView images showing exosomes released from control, NHE9+, and NHE9- cells (*white arrows* indicate exosomes). *Lower panel*: graphical representation of the average number of exosomes derived from control, NHE9+, and NHE9- cells, normalized to their cell numbers. Data represent the average of three biological replicates. Error bars indicate SD. ∗*p* < 0.05, ∗∗*p* < 0.005; statistical analysis performed using Student’s *t* test. *C*, *upper panel*: representative histograms from ZetaView nanoparticle tracking analysis of exosomes produced by control and NHE9+ cells, showing the total particles per ml (*y*-axis) *versus* size in nanometers (*x*-axis). *Lower panel*: graphical representation of the average diameter of exosomes produced by control and NHE9+ cells, based on three biological replicates. Error bars represent SD. NSS, not statistically significant. Statistical analysis was performed using Student’s *t* test. *D*, *upper panel*: representative immunoblot showing CD63 expression in exosomes derived from control and NHE9+ cells. Graphical representation of the average band intensity from densitometric scans of immunoblots, normalized to cell count, based on three biological replicates. Error bars indicate SD. ∗∗*p* < 0.005; statistical analysis performed using Student’s *t* test. *E*, same as *D* except for exosomal marker Alix. *F*, *upper panel*: representative ZetaView images showing exosomes released from control and NHE9+ Vero E6 cells (*white arrows* indicate exosomes). *Lower panel*: graphical representation of the average number of exosomes derived from control and NHE9+ Vero E6 cells, normalized to their cell numbers. Data represent the average of three biological replicates. Error bars indicate SD. ∗∗*p* < 0.005; statistical analysis performed using Student’s *t* test. *G*, same as (*F*), but for exosomes produced by U251 cells. NHE9, Na^+^/H^+^ exchanger isoform 9.

**Fig. S1:**
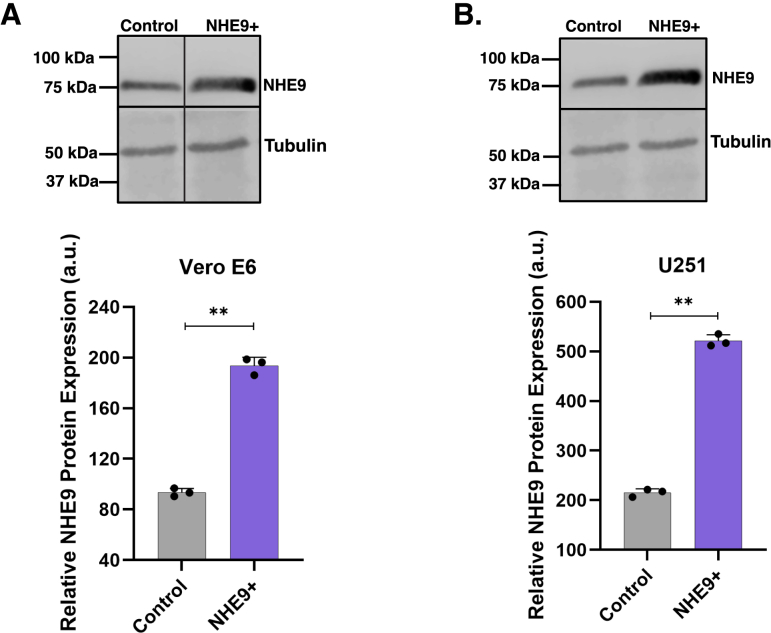


To assess the impact of NHE9 on the production of exosomes, we utilized a lentiviral system to induce overexpression of NHE9 in control HEK293T cells, creating the NHE9+ stable line. This approach yielded a ∼2.59-fold increase in NHE9 protein levels in the NHE9+ cells relative to the control cells (Fig. 1*A*). Remarkably, nanoparticle tracking analysis showed ∼ 141% increase in the number of exosomes released from NHE9+ cells in comparison to the control cells (Fig. 1*B*). To ensure the observed increase in exosome production was specifically due to NHE9 overexpression, we knocked down NHE9 in the NHE9+ cells using shRNA, resulting in a cell line we refer to as NHE9-. The NHE9- cells displayed NHE9 expression levels that were ∼4.25-fold lower than those of NHE9+ cells and ∼1.64-fold lower than those of the unaltered control cells (Fig. 1*A*). In line with a positive correlation between NHE9 expression and exosome production, exosome output in NHE9- cells was reduced by 63% compared to NHE9+ cells and by 12.61% compared to control cells (Fig. 1*B*). Given that NHE9 overexpression significantly enhances exosome production, we will focus on the NHE9 overexpression model from here on to elucidate the mechanisms underlying exosome biogenesis. Analysis of exosome preparations from the supernatants showed a size distribution profile indicating a physically homogeneous population of vesicles peaking at ∼100 nm (Fig. 1*C*-inset). No significant differences in size distribution were associated with changes in NHE9 expression (Fig. 1*C*). Western blot analysis confirmed that the exosome fraction expressed CD63 (Fig. 1*D*) and Alix (Fig. 1*E*), well-established markers of exosomes (19). To ensure that our findings were not unique to HEK293T cells, we established NHE9 overexpression in two additional cell lines: Vero E6, kidney epithelial cells derived from an African green monkey, and U251, human glioblastoma cells (Fig. S1, *A* and *B*). This approach allows us to validate the relevance and robustness of our observations across different species and cell types. Although baseline exosome production can differ significantly between cell lines, we compared exosome output relative to internal controls within each cell line where NHE9 overexpression was induced, rather than making direct comparisons between HEK293T, Vero E6, and U251 cells. Our results were consistent across these models, showing a similar increase in exosome output in both Vero E6 and U251 cells with NHE9 overexpression (Fig. 1, *F* and *G*). These observations provide strong evidence that increased NHE9 expression is associated with elevated exosome production.

### ILV budding is not affected by NHE9

**Figure 2:**
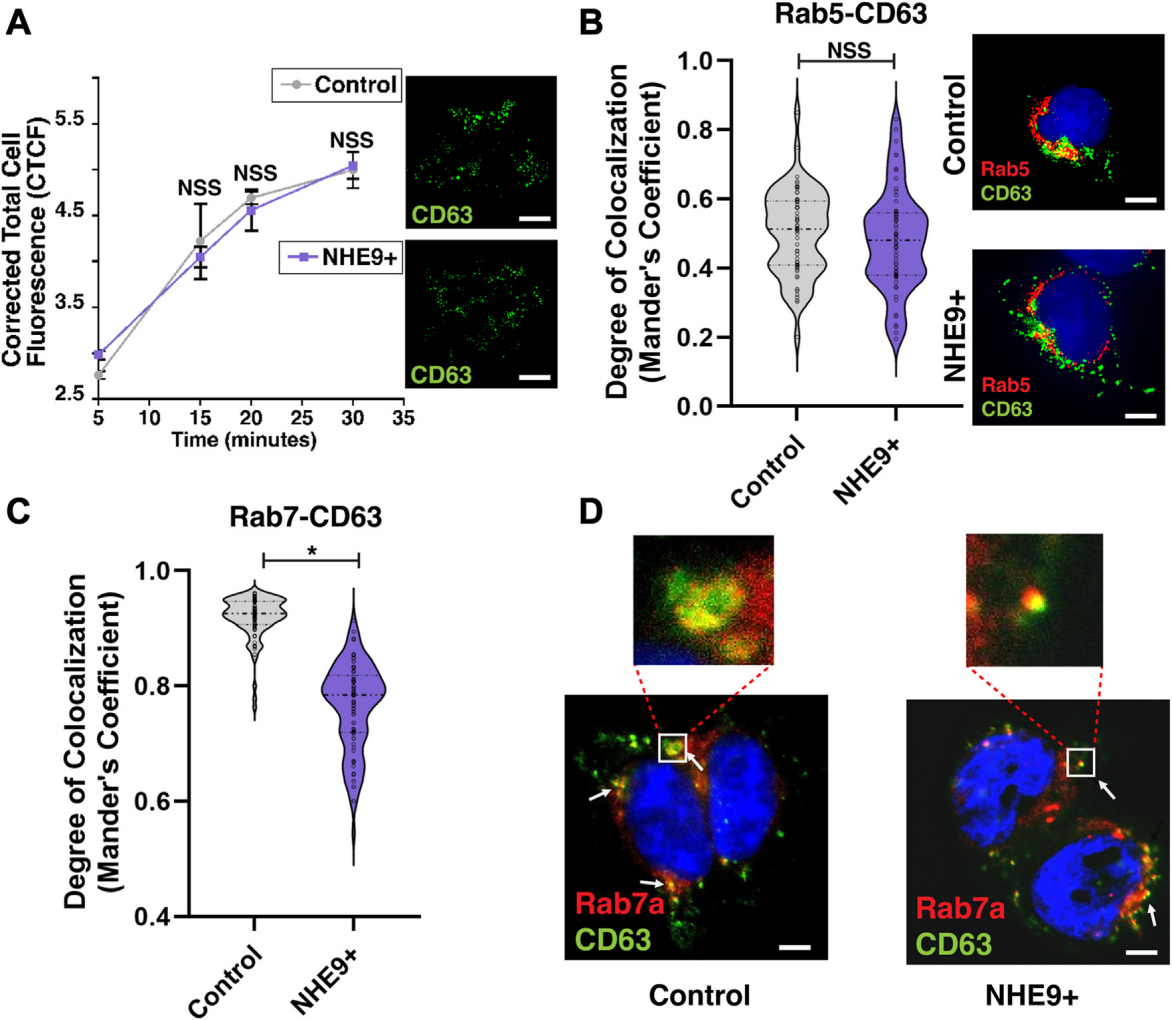
**NHE9 expression does not significantly alter trafficking of exosomal marker CD63 to endosomal compartments.***A*, *left panel*: the graph compares the corrected total cell fluorescence (CTCF) of internalized anti-CD63 antibody between control and NHE9+ HEK293T cells at 5, 15, 20, and 30 min postinternalization. Data represent the average of three biological replicates. Error bars indicate SD. NSS, not statistically significant. Statistical analysis was performed using Student’s *t* test. *Right panel*: representative immunofluorescence microscopy images comparing anti-CD63 antibody (*green*) in control and NHE9+ cells after 20 min of internalization. The scale bar represents 40 μm. *B*, *left panel*: violin plot of Manders’ overlap coefficients (MOCs) comparing the colocalization of Rab5 and CD63 in control and NHE9+ HEK293T cells. Data represent three biological replicates. Error bars indicate SD. NSS, not statistically significant. Statistical analysis was performed using Student’s *t* test. *Right panel*: representative immunofluorescent images depicting the colocalization of the early endosomal marker Rab5 (*red*) and the exosomal marker CD63 (*green*) in control and NHE9+ cells. Colocalization is shown by *yellow regions*. The scale bar represents 10 μm. *C*, violin plot of MOCs) comparing the colocalization of Rab7 and CD63 in control and NHE9+ HEK293T cells. Data represent three biological replicates. Error bars indicate SD. ∗*p* < 0.05; statistical analysis was performed using Student’s *t* test. *D*, representative immunofluorescent images depicting the colocalization of the late endosomal marker Rab7 (*red*) and the exosomal marker CD63 (*green*) in control and NHE9+ cells. Colocalization is shown by *yellow regions*. The scale bar represents 5 μm. NHE9, Na^+^/H^+^ exchanger isoform 9.

**Figure 3:**
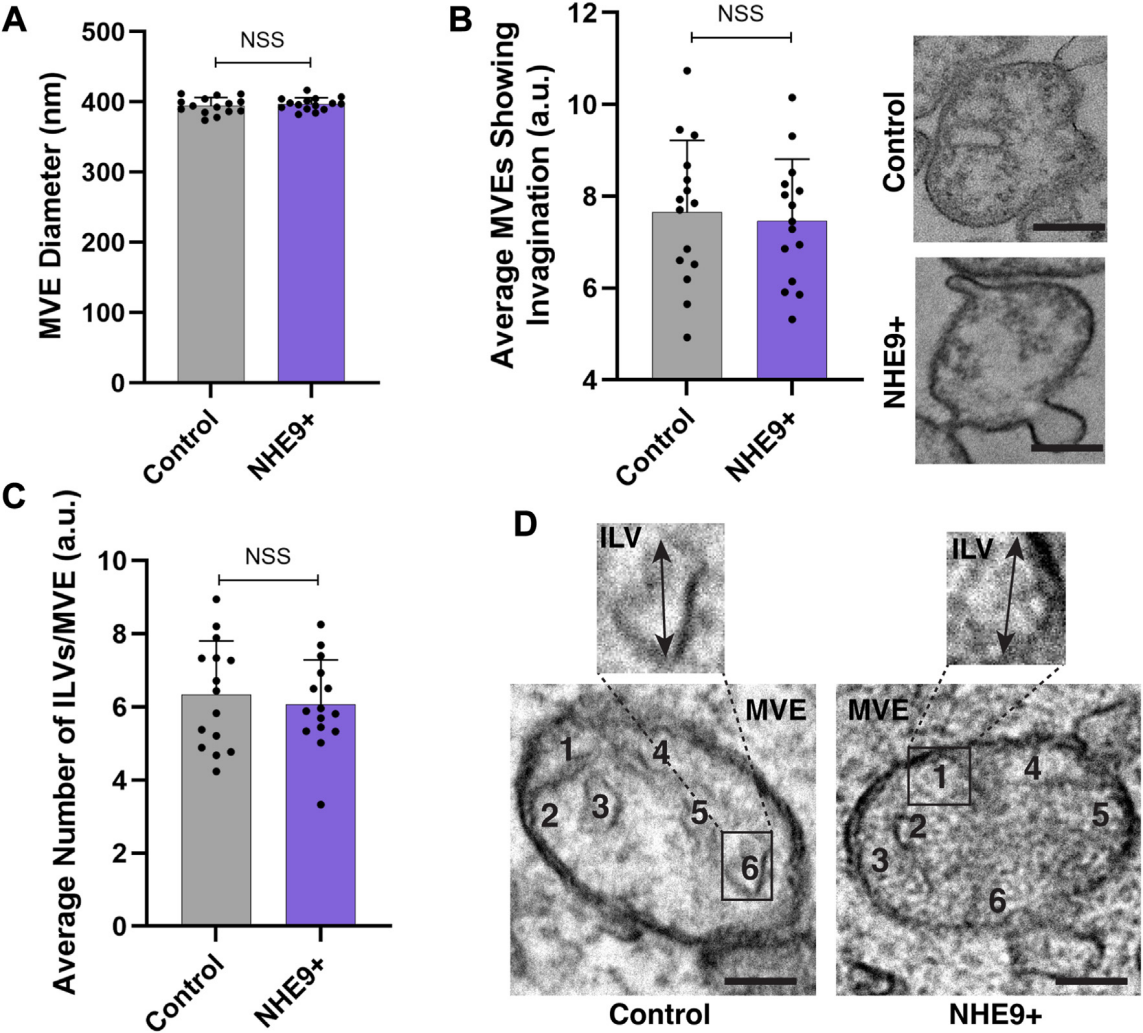
**NHE9 expression does not significantly alter MVE formation and ILV budding.***A*, the graph represents the average multivesicular endosome (MVE) diameter in control and NHE9+ HEK293T cells, as measured by electron microscopy from three biological replicates. Error bars indicate SD. NSS, not statistically significant. Statistical analysis was performed using Student’s *t* test. *B*, *left panel*: the graph represents the average number of MVEs showing membrane invagination in control and NHE9+ cells, as measured by electron microscopy from three biological replicates. Error bars indicate SD. NSS, not statistically significant. Statistical analysis was performed using Student’s *t* test. *Right panel*: representative electron microscopy images comparing MVEs from control and NHE9+ cells. The scale bar represents 200 nm. *C*, the graph represents the average number ofintraluminal vesicles (ILVs) per MVE in control and NHE9+ HEK293T cells, as measured by electron microscopy from three biological replicates. Error bars indicate SD. NSS, not statistically significant. Statistical analysis was performed using Student’s *t* test. *D*, representative electron microscopy images comparing the morphology of ILVs and MVEs in control and NHE9+ HEK293T cells. The scale bar represents 200 nm. NHE9, Na^+^/H^+^ exchanger isoform 9.

To determine the specific step(s) of exosome biogenesis affected by increased NHE9, we first tracked the internalization and subsequent transport of CD63 from the plasma membrane to early and late endosomes. Immunofluorescence analysis of CD63-bound antibody within cells at regular time points after internalization revealed that overexpression of NHE9 did not significantly alter the internalization and transport of CD63 (Fig. 2*A*). We next examined the distribution of CD63 in NHE9+ cells using immunofluorescence microscopy with specific endosome markers (20). In both control and NHE9+ cells, CD63 shows a similar punctate distribution and colocalizes partially with the early endosomal marker Rab5 (Fig. 2*B*). However, while CD63 also colocalizes with the late endosomal marker Rab7, this colocalization is less pronounced in NHE9+ cells compared to control cells (Fig. 2, *C* and *D*). These results suggest that NHE9 expression does not significantly affect the trafficking of exosomal cargo to early and late endosomal compartments. This transport is crucial for ILV development within MVEs. This process begins with the inward budding of the endosomal membrane in MVEs, encapsulating cargo destined for exosomes (1). To investigate the role of NHE9 in ILV budding, we conducted electron microscopy analysis. The MVEs in NHE9+ cells were similar in size (∼400 nm) to those in control cells (Fig. 3*A*). Moreover, the number of MVEs showing membrane invaginations, indicative of MVE budding and scission activity (8), was not significantly different in NHE9+ cells (Fig. 3*B*). Similarly, the average number of ILVs per MVE was consistent (Fig. 3, *C* and *D*). These data clearly demonstrate that NHE9 does not significantly impact the process of MVE formation and ILV budding.

### Alkalization of late endosomes disrupts lysosomal trafficking and promotes exosome biogenesis

**Figure 4:**
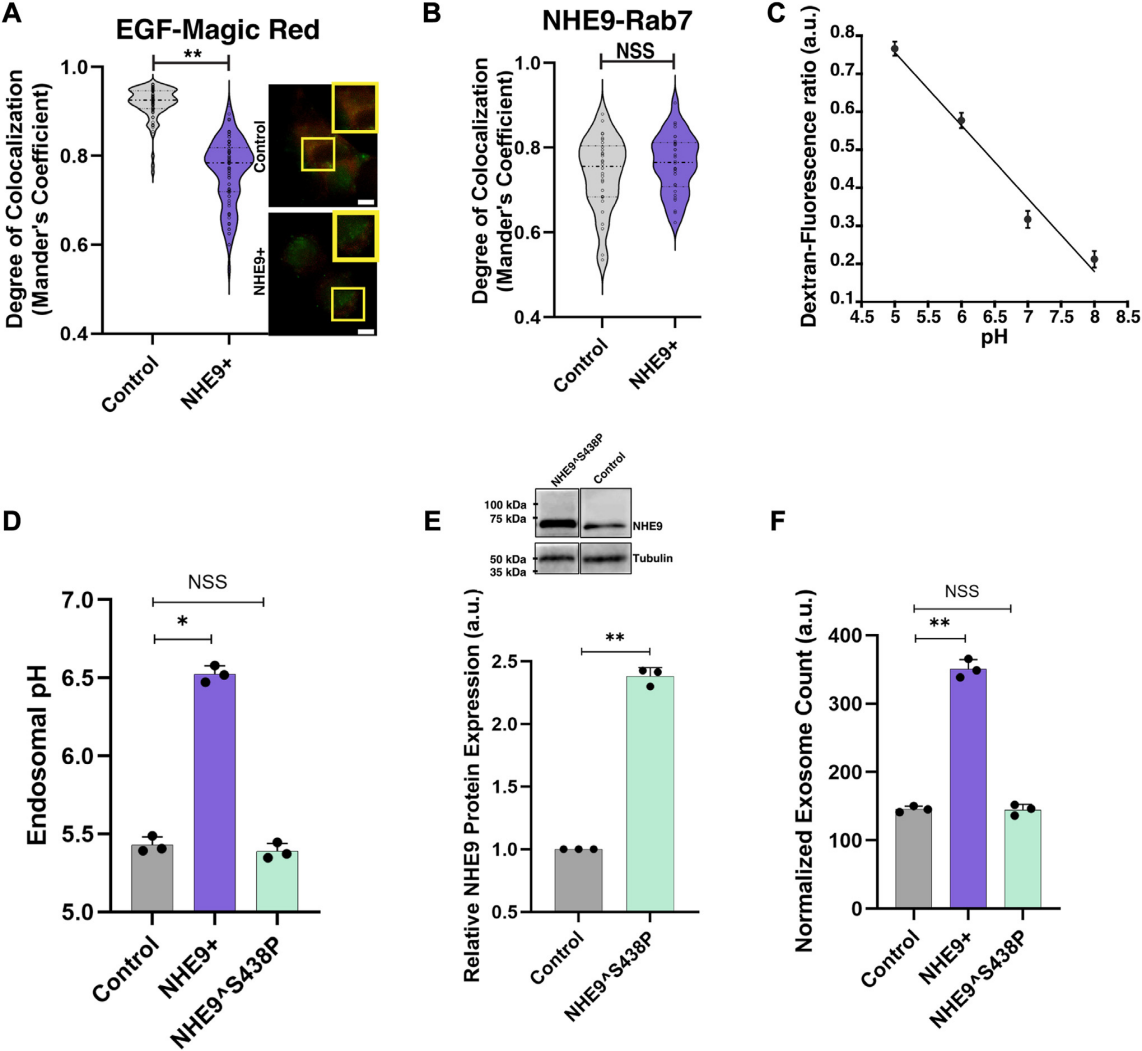
**Alkalization of late endosomes disrupts lysosome trafficking and increases exosome output.***A*, *left panel*: violin plot of Manders’ overlap coefficients (MOCs) comparing the colocalization of epidermal growth factor (EGF) and Magic Red in control and NHE9+ HEK293T cells. Data represent three biological replicates. Error bars indicate SD. Statistical analysis was performed using Student’s *t* test. ∗∗*p* < 0.005. *Right panel*: representative immunofluorescent images depicting the colocalization of EGF (*green*) and Magic Red (*red*) in control and NHE9+ HEK293T cells. Colocalization is shown by *yellow regions*. The scale bar represents 5 μm. *B*, violin plot of MOC comparing the colocalization of NHE9 and the late endosomal marker Rab7 in control and NHE9+ HEK293T cells. Data represent three biological replicates. Error bars indicate SD. NSS, not statistically significant. Statistical analysis was performed using Student’s *t* test. *C*, calibration of late endosomal pH in control HEK293T cells. Cells were loaded with dextran-pHrodo Green and dextran-Alexa Fluor 568, then exposed to nigericin, and assayed in pH-defined medium (pH 5.0–pH 8.0) to calculate their fluorescence ratio. *D*, graph comparing pH in dextran-positive late endosomal compartments between control, NHE9+, and functional NHE9 mutant (NHE9ˆS438P) HEK293T cells. Data represent the average of three biological replicates. Error bars indicate SD. Statistical analysis was performed using Student’s *t* test. ∗*p* < 0.05. *E*, *lower panel*: graphical representation of the average band intensity from densitometric scans of immunoblots showing NHE9 expression in control and NHE9ˆS438P HEK293T cells from three biological replicates. Error bars indicate SD. Statistical analysis was performed using Student’s *t* test. ∗∗*p* < 0.005. *Upper panel*: representative immunoblot showing NHE9 expression in control and NHE9ˆS438P HEK293T cells. *F*, graphical representation of the average number of exosomes produced by control, NHE9+, and NHE9ˆS438P HEK293T cells, normalized to their cell numbers. Data represent the average of three biological replicates. Error bars indicate SD. NSS: Not statistically significant. ∗∗*p* < 0.005; statistical analysis performed using Student’s *t* test. NHE9, Na^+^/H^+^ exchanger isoform 9.

**Fig. S2:**
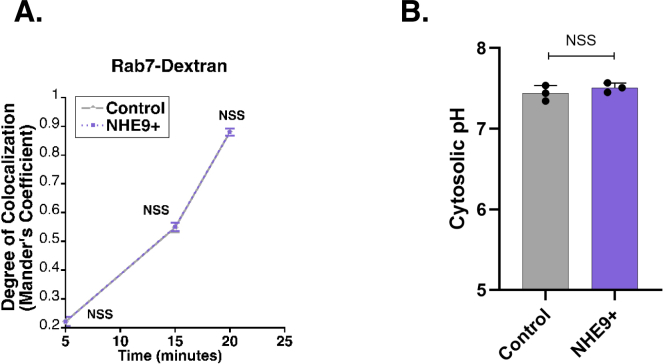


NHE9 is postulated to function as a proton leak pathway, thereby moderating the acidification of the endosomal lumen by the proton-pumping V-ATPase (3, 4, 21). While CD63 colocalizes with both Rab5 and Rab7 in control and NHE9+ cells, it is noteworthy that the degree of colocalization with Rab7 is significantly reduced in NHE9+ cells, as shown in Figure 2, *C* and *D*. Considering the critical role of late endosomes in the trafficking of exosome cargo, we hypothesized that the diminished colocalization between Rab7 and CD63 in NHE9+ cells could indicate a disruption in the normal trafficking pathway. This disruption might potentially result in less delivery of cargo to the lysosome. To probe this, we employed the Magic Red (MR) cathepsin assay (22). The MR compound, which is not fluorescent on its own, acts as a substrate for cathepsins within the lysosomes and emits a red fluorescent signal upon cleavage by these active enzymes. We compared the colocalization of green fluorescence–tagged epidermal growth factor (EGF) with the red fluorescence from MR in both NHE9+ and control cells. Consistent with our hypothesis, NHE9+ cells showed an approximate 72% reduction in EGF delivery to lysosomes compared to control cells (Fig. 4*A*). Based on our previous studies in macrophages showing that endosome alkalization mediated by NHE9 underlies trafficking defects to the lysosome, we measured the luminal pH of late endosomes (5). To this end, we first confirmed that NHE9 localization to late endosomes did not change because of NHE9 overexpression (Fig. 4*B*). To assess the luminal pH of late endosomes (pH_le_), we applied ratiometric imaging with pH-sensitive (pHrodo Green–conjugated) and pH-insensitive (Alexa Fluor 568–conjugated) dextrans (23). The pH-insensitive fluorescence serves to normalize for differences in endosomal uptake. These dextrans are rapidly internalized by endocytosis and accumulate in late endosomes within 20 min in both control and NHE9+ cells (Fig. S2*A*). In NHE9+ cells, luminal pH measurements showed an elevation approximately one pH unit (Fig. 4, *C* and *D*). In contrast, cytoplasmic pH remained unchanged (Fig. S2*B*). We investigated how luminal acidification affects exosome production by evaluating the impact of overexpressing the NHE9 mutant S438P, which has a loss of function in pH regulation (16). Although this mutant was expressed at levels similar to the WT in control cells (Figs. 1*A* and 4*E*), cells harboring the NHE9^∧^S438P mutant did not show a significant change in pH_le_ compared to the control cells (Fig. 4*D*). In line with our hypothesis, nanoparticle tracking analysis revealed no statistically significant difference in exosome production between control cells and those expressing the functional mutant, as shown in Figure 4*F*. Together, these findings demonstrate that the luminal pH of late endosomes regulates exosome production, with alkalization leading to disruption in lysosomal trafficking and an increase in exosome output.

### Rab7 activation underlies the effect of luminal pH on exosome biogenesis

**Figure 5:**
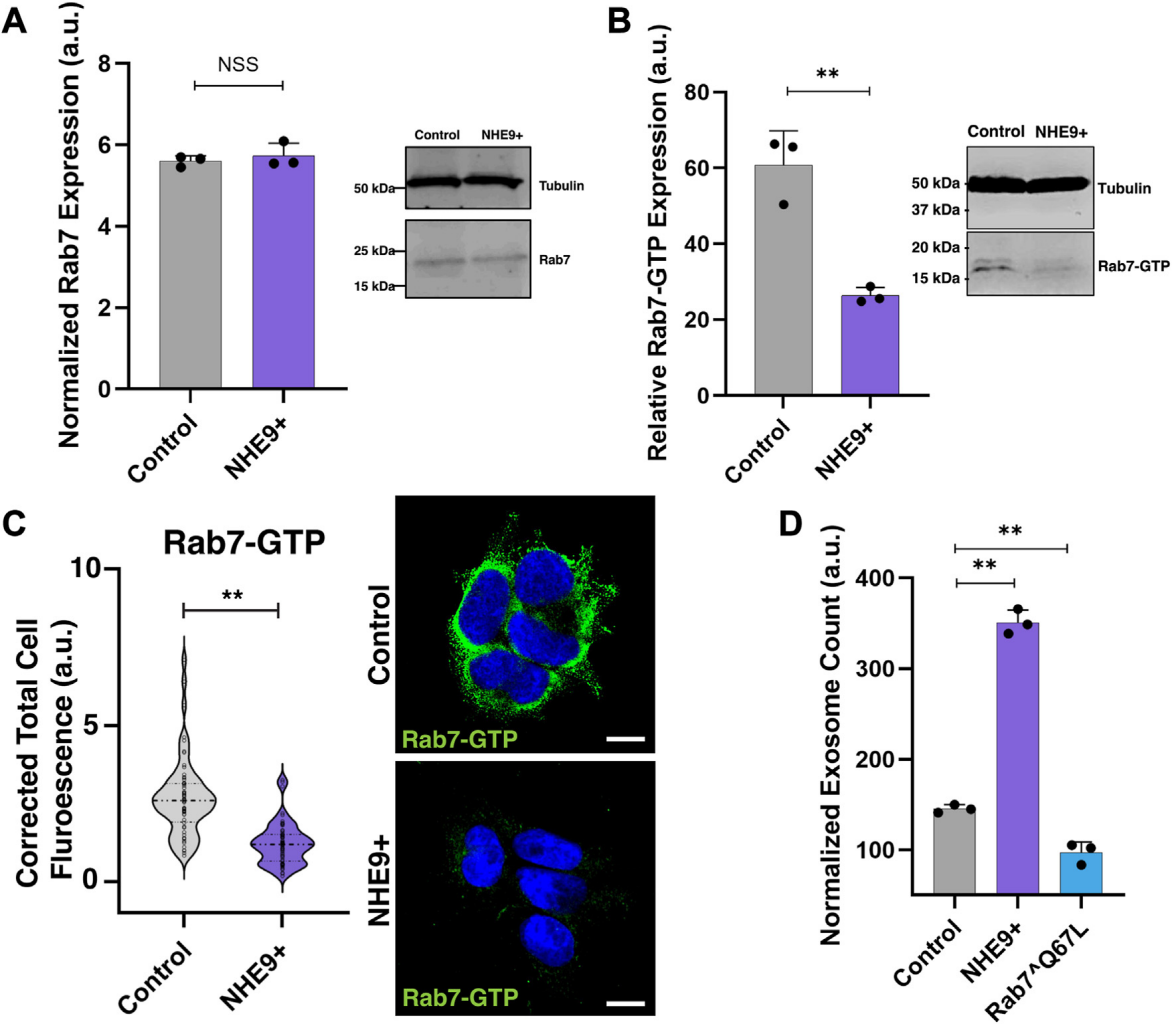
**Rab7 activation links endosomal acidification to exosome biogenesis.***A*, *left panel*: graphical representation of the average band intensity from densitometric scans of immunoblots showing Rab7 expression in control and NHE9+ HEK293T cells from three biological replicates. Error bars indicate SD. NSS, not statistically significant. Statistical analysis was performed using Student’s *t* test. *Right panel*: immunoblot showing Rab7 expression in control and NHE9+ HEK293T cells. *B*, *left panel*: graphical representation of the average band intensity from densitometric scans of immunoblots showing active Rab7 (*i.e.*, Rab7-GTP) expression in control and NHE9+ HEK293T cells from three biological replicates. Error bars indicate SD. ∗∗*p* < 0.005; statistical analysis was performed using Student’s *t* test. *Right panel*: representative immunoblot showing active Rab7 (*i.e.*, Rab7-GTP) expression in control and NHE9+ HEK293T cells. *C*, *left panel*: violin plots of corrected total cellular fluorescence comparing Rab7-GTP expression in control and NHE9+ HEK293T cells based on indirect immunofluorescence using a Rab7-GTP antibody. Data represent three biological replicates. Error bars indicate SD. Statistical analysis was performed using Student’s *t* test. ∗∗*p* < 0.005. *Right panel*: representative immunofluorescence images depicting Rab7-GTP expression (*green*) and its localization with respect to 4′,6-diamidino-2-phenylindole (*blue*) in control and NHE9+ HEK293T cells. The scale bar represents 5 μm. *D*, graphical representation of the average number of exosomes produced by control HEK293T, NHE9+, HEK293T, and NHE9+ HEK293T cells expressing the constitutively active Rab7 mutant (Rab7ˆQ67L), normalized to their cell numbers. Data represent the average of three biological replicates. Error bars indicate SD. ∗∗*p* < 0.005; statistical analysis was performed using Student’s *t* test. NHE9, Na^+^/H^+^ exchanger isoform 9.

To elucidate the mechanism linking hindered lysosomal cargo delivery to luminal alkalization, we investigated whether luminal pH affects the overall intracellular expression of Rab7. Given Rab7's critical role in recruiting motor proteins for late endosomal trafficking, we hypothesized that alterations in luminal pH might influence Rab7 levels (24). However, Western blot analysis revealed no significant difference in total Rab7 levels between control and NHE9+ cells (Fig. 5*A*). Rab7, a key GTPase in its active, GTP-bound state, mediates the transport of MVEs to lysosomes by recruiting the dynein–dynactin complex through Rab-interacting lysosomal protein (24). Considering this, we next investigated whether impaired acidification in NHE9+ cells was associated with changes in the levels of active Rab7 rather than its overall expression. To test this hypothesis, we conducted Western blot analysis using antibodies that specifically recognize the GTP-bound form of Rab7, comparing its levels between control and NHE9+ cells. Our results revealed an ∼2.2-fold decrease in Rab7-GTP in NHE9+ cells compared to controls (Fig. 5*B*). These observations are further supported by immunofluorescence microscopy, which showed a striking decrease in Rab7-GTP fluorescence intensity in NHE9+ cells (Fig. 5*C*). To confirm whether the increase in exosome production in NHE9+ cells is a consequence of reduced active Rab7 levels, we ectopically expressed a constitutively active Rab7 mutant (Q67L) in NHE9+ cells (Fig. S3) (25). Remarkably, expressing Rab7^∧^Q67L reversed the exosomal increase observed in NHE9+ cells, as shown in Figure 5*D*. These findings clearly indicate that the reduced levels of active Rab7 in NHE9+ cells contribute to increased exosome production. This highlights Rab7 activation as a key mechanism connecting luminal acidification to exosome biogenesis.

### Limiting endosomal pH enhances Rab27b association and plasma membrane fusion of MVEs

**Figure 6:**
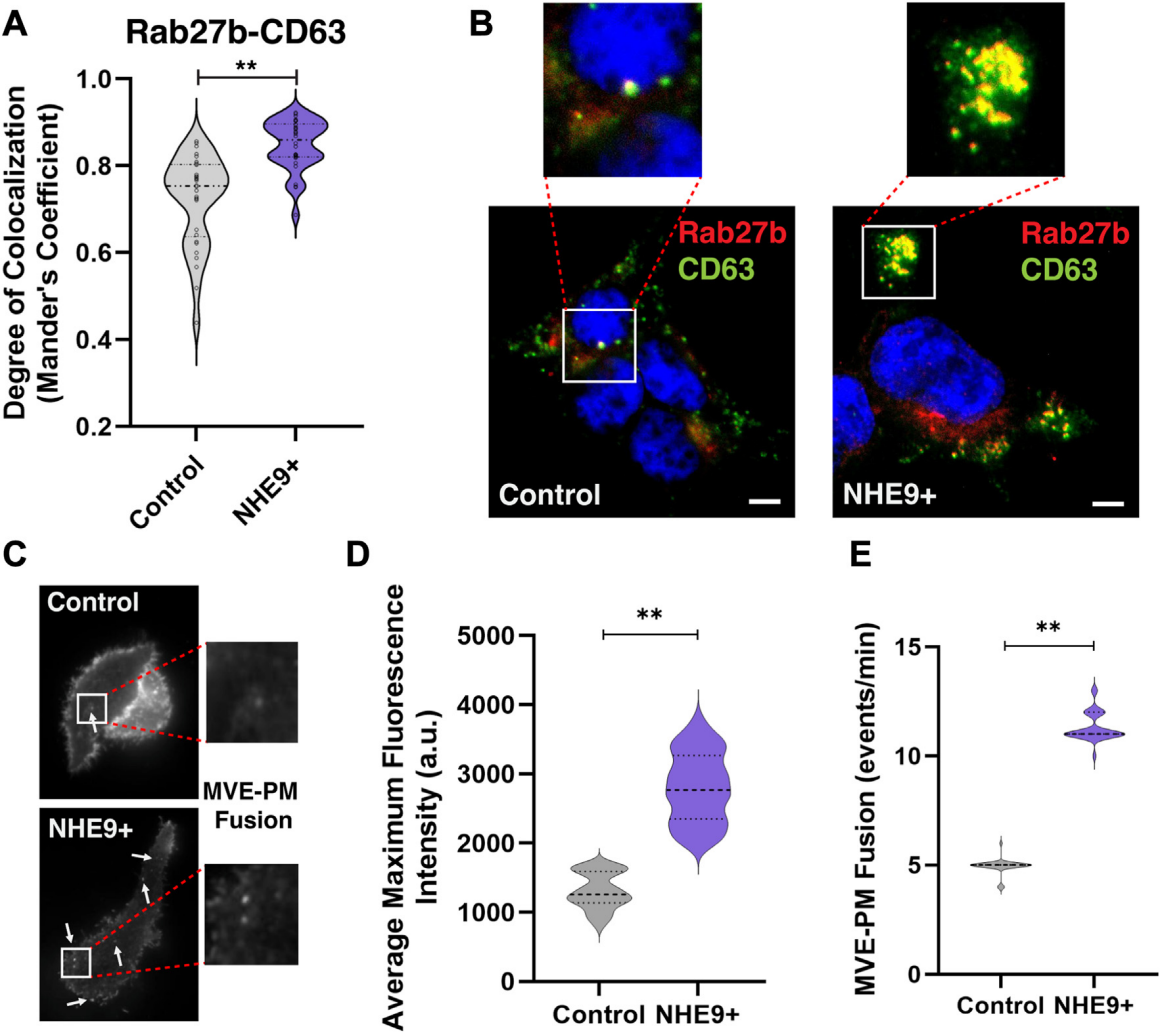
**Reduced endosomal acidification promotes Rab27b association and MVE-PM fusion.***A*, violin plot of Manders’ overlap coefficients (MOCs) comparing the colocalization of Rab7 and CD63 in control and NHE9+ HEK293T cells. Data represent three biological replicates. Error bars indicate SD. Statistical analysis was performed using Student’s *t* test. ∗∗*p* < 0.005. *B*, representative immunofluorescent images depicting the colocalization of Rab27b (*red*) and CD63 (*green*) in control and NHE9+ HEK293T cells. Colocalization is shown by *yellow regions*. The scale bar represents 5 μm. *C*, representative total internal reflection fluorescence (TIRF) microscopy images using the pH-sensitive fluorescent reporter CD63-pHluorin, comparing multivesicular endosomes (MVEs) and plasma membrane (PM) fusion events between control and NHE9+ HEK293T cells. *D*, graphical representation comparing the maximum fluorescence intensity of MVE-PM fusion events in control and NHE9+ HEK293T cells. Data represent the average of three biological replicates. Error bars indicate SD. ∗∗*p* < 0.005. Statistical analysis was performed using Student’s *t* test. *E*, graphical representation comparing the number of MVE-PM fusion events per minute in control and NHE9+ HEK293T cells. Data represent the average of three biological replicates. Error bars indicate SD. ∗∗*p* < 0.005. Statistical analysis was performed using Student’s *t* test. NHE9, Na^+^/H^+^ exchanger isoform 9.

While the loss of Rab7 activity explains the disruption in lysosomal cargo delivery, it does not completely account for the increased trafficking to the plasma membrane for exosomal secretion. To this end, we investigated whether NHE9+ cells show a greater association of Rab27b with MVEs. Rab27b is a small GTPase involved in directing the secretion of MVE cargo, possibly by facilitating the connection between MVEs and outbound motor proteins (26). We utilized immunofluorescence microscopy to examine the colocalization of Rab27b with the MVE marker CD63 in both control and NHE9+ cells. Our results indicate approximately a 24% increase in the colocalization of Rab27b and CD63 in the NHE9+ cells compared to control cells (0.92 ± 0.005 *versus* 0.74 ± 0.045) (Fig. 6, *A* and *B*). Next we investigated if this enhanced association led to more MVE fusion events at the plasma membrane. To this end, we employed live imaging with a tetraspanin-based, pH-sensitive fluorescent reporter (CD63-pHluorin) to quantify the fusion rate of MVEs with the plasma membrane (PM) in single cells (27). The fluorescence is quenched in the acidic lumen of MVEs. However, upon fusion with the PM, the low luminal pH is quickly neutralized, leading to a rapid increase in fluorescence intensity detectable by live total internal reflection fluorescence (TIRF) microscopy (Videos S1 and S2). A complete MVE–PM fusion event is characterized by an immediate increase in fluorescence intensity, resulting from the rapid neutralization of MVE pH during exocytosis and the subsequent movement of secreted exosomes into the view of the TIRF microscopy field. This fluorescence peak typically diminishes exponentially, remaining stable at one location for at least 2 s. The average fluorescence intensity upon PM fusion was ∼2.3-fold higher in NHE9+ cells relative to control cells (Fig. 6, *C* and *D*). Not all fluorescence increases indicate exosome release, as some originate from incomplete fusion events that do not lead to exosomal secretion (27). Through careful examination of fluorescence patterns before and after the initial spike based on previously published protocols (27), we successfully differentiated between full MVE–PM fusions and nonproductive events in our live imaging studies. We noted an ∼2-fold increase in full MVE-PM fusion events in NHE9+ cells compared to control cells (Fig. 6*E*). These data clearly demonstrate a notable shift toward the exosomal pathway when Rab7 activity is reduced, as evidenced by an increased association with Rab27b and a corresponding rise in the number of fusion events at the PM.

## Discussion

Our study elucidates the pivotal role of endosomal pH regulation in exosome biogenesis and secretion. We observed a significant increase in exosome output with NHE9-mediated alkalization across diverse cell lines. This consistency underscores the robustness of NHE9’s effect on exosome biogenesis and suggests a mechanism applicable across various cellular contexts. The NHE9-knockdown experiments, which demonstrated a reduction in exosome production, further solidify the positive correlation between NHE9 expression and exosome output. These findings position NHE9 as an important regulator of exosome biogenesis and align with recent discoveries that disruptions in V-ATPase activity which prevent normal MVE acidification, increase exosome secretion (8).

In support of our findings, previous studies in yeast have highlighted the importance of pH regulation in vesicular trafficking. Notably, Nhx1, the yeast equivalent of the mammalian NHE9, has been shown to interact with Gyp6, which acts as a GTPase-activating protein for Ypt6, a GTPase crucial for vesicle trafficking (28). This suggests that Nhx1 might affect the balance between anterograde and retrograde trafficking, akin to how NHE9-mediated alkalization affects endosomal trafficking in mammalian cells. Furthermore, Kallay *et al.* found that MVE formation is not disrupted in *nhx1* KO yeast (29). This finding parallels our observation in Figure 3, which shows that changes in NHE9 do not significantly impact MVE formation and ILV budding in mammalian cells. Although the specific molecular players and mechanisms may differ between yeast and mammalian systems, these findings collectively emphasize the conserved role of pH regulation in modulating vesicular trafficking across eukaryotic organisms.

**Figure 7:**
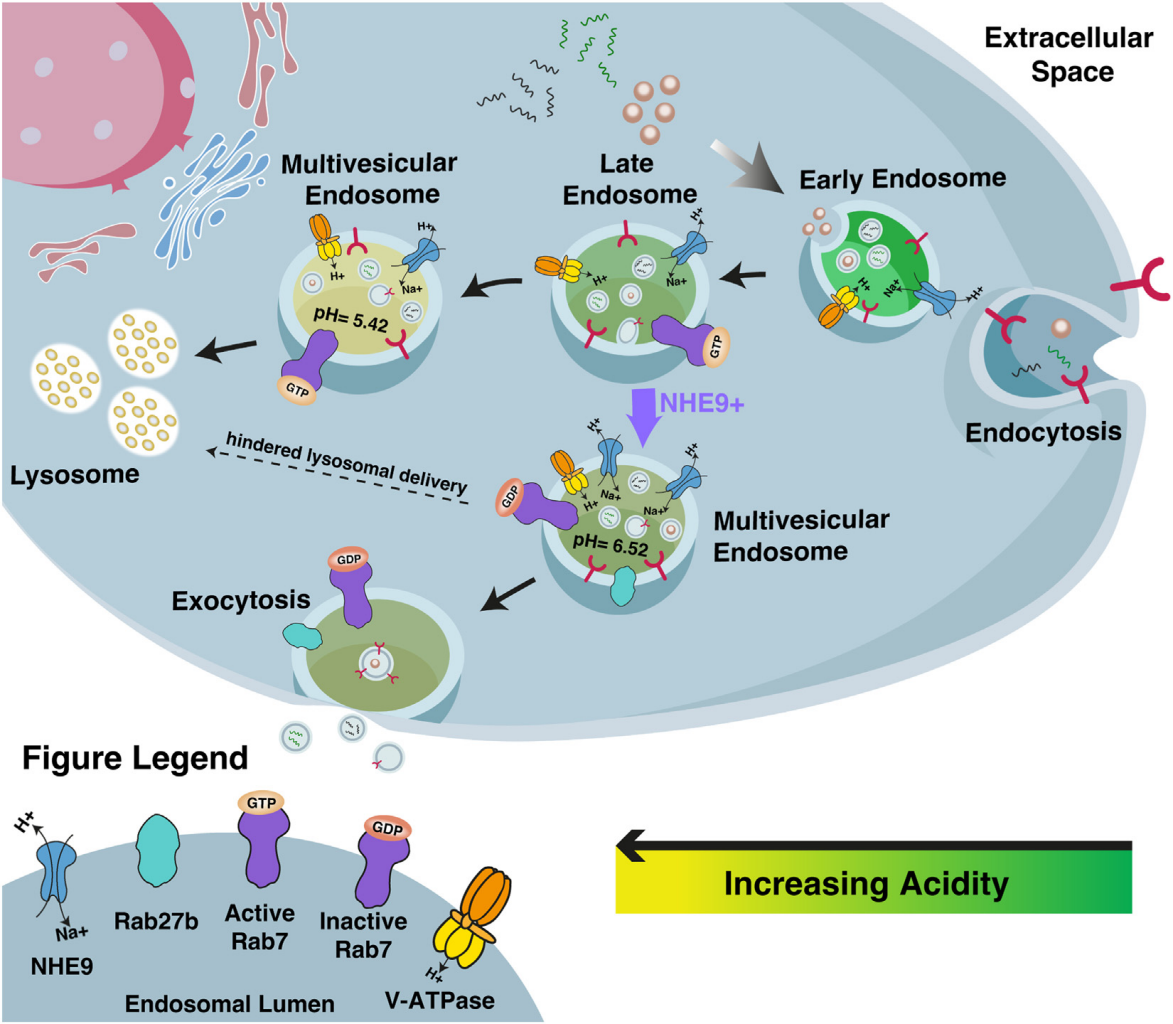
**Schematic illustrating the mechanism by which NHE9-mediated endosomal pH regulation impacts exosome biogenesis and secretion.***A*, NHE9 fine-tunes endosomal acidification by facilitating the exchange of protons out of the endosome with Na+ or K+. Increased NHE9 expression leads to endosomal alkalinization due to proton leakage. This alkalinization impairs Rab7 activation, disrupting the delivery of multivesicular endosomes (MVEs)to lysosomes. Instead, it promotes the recruitment of Rab27b to MVEs, directing them to the plasma membrane and enhancing exosome secretion. NHE9, Na^+^/H^+^ exchanger isoform.9

Mechanistically, we demonstrate that the activation of Rab7 links NHE9-mediated alterations in endosomal pH to exosome biogenesis (Fig. 7). Specifically, NHE9-induced endosomal alkalization impairs Rab7 activation, consequently hindering the transport of MVEs to lysosomes. Constitutively active Rab7 rescues exosome secretion in NHE9-overexpressing cells, restoring it to levels comparable to those in WT cells. This underscores the critical role of Rab7 activation in mediating NHE9's effects on endosomal trafficking. Interestingly, a previous study using Nhx1, found no effect of its deletion on the activation state of Ypt7, the yeast ortholog of Rab7 (30). However, this study did not investigate the impact of alkalization. The discrepancy between our results and those of the yeast studies could be due to species-specific regulatory adaptations. These differences could result in distinct compensatory mechanisms or regulatory controls governing Rab GTPase activity. It is possible that yeast cells possess alternative pathways that sustain Ypt7 activation even in the absence of Nhx1, suggesting potential redundancies in their vesicular trafficking systems. In contrast, in mammalian cells, NHE9's influence appears to be more direct and less compensated, indicating a critical dependency on endosomal pH for Rab7 function and subsequent exosome biogenesis. We note that findings in yeast suggesting issues with the fusion of MVBs with the vacuole/lysosome when late endosomal pH is altered could still be applicable to mammalian cells (30). We have not directly tested this in mammalian cells. Our data clearly highlights the problem of Rab7 activation on MVBs, which occurs upstream of their fusion with the lysosome. The specific mechanisms through which luminal pH impacts Rab7 activity remain to be determined.

While NHE9-mediated endosomal pH alkalization impairs the trafficking of MVEs to lysosomes, it also promotes exosome secretion by directing MVEs to the cell periphery (Fig. 7). Rab27b plays a crucial role in this process by transporting MVEs along microtubules to the actin-rich cortex and facilitating their docking at the cell periphery (26). We demonstrate that NHE9-mediated alkalization increases the recruitment of Rab27b to MVEs, resulting in enhanced PM fusions. The exact mechanism underlying this recruitment remains unknown. Recent studies have indicated that an octameric protein complex known as the exocyst is essential for directing MVEs to the cell periphery (31). The exocyst interacts with SNARE proteins and the Rab family of GTPases, facilitating the trafficking, docking, and fusion of MVEs with the PM (31, 32, 33). Given that endosomal pH can influence protein interactions on vesicular membranes (34), it is plausible that NHE9-mediated alkalization enhances the recruitment and activity of both the exocyst complex and Rab27b, thereby promoting efficient exosome secretion.

Our findings highlight the potential of NHE9 as a regulatory mechanism for applications where control over exosome numbers is essential. By modulating endosomal pH through NHE9, it is possible to increase exosome production, which could be beneficial in therapeutic settings such as drug delivery, where higher exosome yield is desirable. Conversely, reducing NHE9 activity could help in conditions where minimizing exosome-mediated communication is beneficial. While our study has demonstrated that NHE9-mediated alkalization increases the number of exosomes, it remains to be determined whether the content and functional activity of these exosomes is also affected by changes in NHE9 levels. Future research should investigate these aspects to fully understand the implications of NHE9 modulation on exosome-based therapies.

## Experimental procedures

### Cell lines and plasmids

Cultures of HEK293T, Vero E6 (from BEI Resources), and U251 cells were maintained in Dulbecco's Modified Eagle's Medium (; Thermo Fisher Scientific) supplemented with 10% fetal bovine serum (Sigma) and 5% antibiotic-antimycotic solution (10,000 units/ml penicillin, 10,000 mg/ml streptomycin; Gibco). Cells were cultured fewer than 5% CO2 at 37 °C. The NHE9+ and the functional mutant S438P (NHE9+ ^∧^S438P) were stably overexpressed in HEK293T cells through transfection using Lipofectamine 3000 (Invitrogen), followed by selection with 10 μg/ml blasticidin. NHE9 knockdown (NHE9-) and constitutively active Rab7 (Rab7^∧^Q67L) were stably expressed in NHE9+ cells through transfection with Lipofectamine 3000 (Invitrogen). NHE9- was achieved using the shRNA clone pGipZ-SLC9A9-178966 (Horizon Discovery). EGFP-Rab7A Q67L was provided as a gift from Qing Zhong (Addgene plasmid # 28049; RRID:Addgene_28049) (35). Both clones included GFP for selection. NHE9 overexpression in Vero E6 and U251 cells were achieved through lentiviral transduction, followed by selection with 10 μg/ml blasticidin. The ORF expression clone for SLC9A9 (NM_173653.3) was obtained from GeneCopoeia.

### Exosome isolation and ZetaView analysis

Cells were cultured until approximately 70% confluency, then washed with PBS, and incubated in media containing exosome-depleted fetal bovine serum (A2720803, Thermo Fisher Scientific) for 48 h. Postincubation, the media was collected, and cell counts were recorded for normalization purposes. Exosomes were isolated using the total exosome isolation reagent from Invitrogen (CAT# 4478359) according to the manufacturer's instructions.

For particle size distribution and concentration, the ZetaView Nanoparticle Tracking instrument (Particle Metrix) was employed. Exosome samples were diluted in PBS to achieve concentrations within the instrument's measurable range. A 1 ml aliquot of the sample was injected into the device and allowed to decelerate according to the built-in particle drift sensor. When the sample concentration fell within the acceptable range, video acquisition and analysis were performed using the following parameters: acquisition parameters: sensitivity (70), shutter speed (120), and positions measured (11). Background readings from media devoid of exosomes were subtracted to ensure accurate measurement.

### Protein extraction and Western blotting

Cells were harvested at approximately 85% confluency by centrifugation at 1100 rpm for 5 min. The cell pellets were then resuspended in a mammalian protein extraction reagent (Thermo Fisher Scientific, 78501) supplemented with Halt protease and phosphatase inhibitor cocktails (Thermo Fisher Scientific; 87785, 78420). The lysates were further centrifuged at 14,000*g* for 8 min at 4 °C. The supernatants were combined with Laemmli loading buffer (375 mM Tris–HCl, pH 6.8, 9% SDS, 50% glycerol, 9% β-mercaptoethanol, and 0.03% bromophenol blue; Thermo Fisher Scientific) and heated in a 70 °C water bath for 10 min.

The protein samples were separated by SDS-PAGE gel electrophoresis at 100 V for 90 min using Novex Tris-Glycine SDS Running Buffer (Thermo Fisher Scientific, LC2675). Proteins were then transferred to a polyvinylidene difluoride membrane in Tris/Glycine Buffer (25 mM Tris, 192 mM glycine, pH 8.3; Bio-Rad) at 100 V for 90 min at 4 °C. The membrane was blocked at room temperature (RT) for 60 min. It was then incubated overnight at 4 °C with primary antibodies diluted in blocking solution: SLC9A9 antibody (1:100, PA5-42524, ThermoFisher Scientific), CD63 antibody (1:100, H5C6-s, DSHB), Rab7a antibody (1:100, PA5-52369, Thermo Fisher Scientific), Rab7-GTP antibody (1:100, 26923, NewEast Biosciences), and monoclonal α-tubulin antibody (1:500, T6199, Sigma). Primary antibodies were validated using blocking peptides followed by immunofluorescence microscopy or through literature citations where the antibodies were previously utilized. The membrane was washed with TBST (20 mM Tris, 500 mM sodium chloride, pH 7.5, 1% Tween; Bio-Rad) before and after a 60-min incubation at RT with 1:5000 dilutions of IRDye 680RD and IRDye 800CW secondary antibodies in blocking solution (LI-COR; 926-68071, 925-32210). Blots were imaged using the LI-COR Odyssey Fc system and analyzed using ImageJ software (https://imagej.net/ij/download.html).

### Electron microscopy

Cells were initially fixed with 3% glutaraldehyde and 3% formalin in 0.1 M sodium cacodylate buffer (CB) for 1 h at RT. After fixation, the samples were rinsed three times for 15 min each with 0.1 M CB. Postfixation was carried out in a mixture of 1.5% potassium ferrocyanide [K₄Fe(CN)₆] and 2% osmium tetroxide (OsO₄) in 0.1 M CB for 30 min, followed by three additional 5-min washes with 0.1 M CB. The cells were then scraped, pelleted by centrifugation, and embedded in 4% agarose. The pellets underwent three 5-min washes with 0.1 M acetate buffer (AB) and were stained with 2% uranyl acetate in 0.1 M AB for 1 h. Excess uranyl acetate was eliminated by two additional washes in 0.1 M AB (5 min each) and a final 5-min wash with deionized water. The samples were dehydrated in a graded ethanol series (30%, 50%, 70%, 80%, 90%, 95%, 100%, and two final changes of 100% ethanol), each for 15 min. This was followed by an acetone infiltration series, in which the cells were incubated in acetone mixtures (2:1 for 1 h, 1:1 for 2 h, and 1:2 overnight) at RT. Samples were then incubated in 100% resin under vacuum for 24 h and subsequently polymerized at 70 °C for another 24 h.

Imaging was conducted using a JEOL JEM 1400 PLUS transmission electron microscope, equipped with a LaB6 filament, capable of imaging thin samples (<200 nm) with a resolution of 0.38 nm. The microscope operates at accelerating voltages ranging from 40 kV to 120 kV, with a magnification range of 10x to 12,00000x. Images were captured using a 2k CMOS camera, and the system includes a quick-release RT retainer (EM-11610 QR1) allowing for ±20° tilt for sample orientation.

### Immunofluorescence microscopy and image analysis

Cells were grown on Poly-L-Lysine–treated coverslips, washed with PBS, and fixed with 4% paraformaldehyde for 10 min at RT. After washing with cold PBS, the cells were permeabilized with 0.1% Triton-X in PBS containing 1% bovine serum albumin and 0.3 M glycine for 10 min. Following this, the cells were blocked for 1 h in a solution of 1% bovine serum albumin and 0.3 M glycine in PBS.

Cells were then incubated overnight at 4 °C with primary antibodies diluted 1:100 in blocking solution: NHE9 antibody (PA5-42524, Thermo Fisher Scientific), Rab5 antibody (PAS-29022, Thermo Fisher Scientific), Rab7a antibody (PA5-52369, Thermo Fisher Scientific), Rab7-GTP (26923, NewEast Biosciences), or Rab27b antibody (AB0072-200, SICGEN). For colocalization studies, cells were stained with two primary antibodies at 1:100 dilution each.

For uptake assays, cells were treated with EGF-Alexa Fluor 488 (E13345, Invitrogen) and MR (MR-RR2, Bio-Rad), incubated on ice for 30 min, and then transferred to 37 °C. Cells were fixed at various time points post endocytosis. For CD63 internalization assays (36), cells were incubated for 30 min at 4 °C with 2 ug/ml anti-CD63 antibody in complete media, washed twice in PBS, and then incubated at 37 °C. Cells were fixed after internalization at various time points.

After primary antibody treatments, the cells were washed three times with PBS and then incubated with Alexa Fluor–conjugated secondary antibodies (Invitrogen) at 1:500 dilution for 1 h. The cells were washed three more times with PBS, treated with 4′,6-diamidino-2-phenylindole for 5 min, and then washed again with PBS before mounting onto slides using antifade mounting medium (Vectashield, Vector Labs).

Cells were imaged using the epifluorescence setting of the Echo Revolve R4 microscope. At least 30 cells per test group were scanned across three independent experiments. Manders’ overlap coefficients were determined using the Colocalization Finder plugin in ImageJ (37). Fluorescence intensity was measured by calculating the corrected total cell fluorescence value for each cell. This was done by subtracting the product of the cell area and the mean fluorescence of the background reading from the integrated density of the cell.

For live-cell imaging, cells were washed with PBS and transferred to live-cell imaging media (Thermo Fisher Scientific) before imaging with the upright setting of the Echo Revolve R4 microscope. All image analyses were performed using ImageJ software.

### pH measurements

Cells were rinsed with PBS and incubated in serum-free medium for 5 min. Following this, the cells were exposed to 70 μg/ml of dextran conjugated to a pH-sensitive fluorophore (pHrodo Green, Thermo Fisher Scientific) and 70 μg/ml of dextran conjugated to a pH-insensitive fluorophore (Alexa Fluor 568, Thermo Fisher Scientific) for 30 min at 37 °C. Endocytosis was halted by placing the cells on ice. Excess dextran was removed by washing with ice-cold PBS.

The fluorescence intensities of at least 5000 cells were measured using the Bio-Rad ZE5 flow cytometer, and the average intensity of the cell population was recorded (3). A pH calibration curve was generated by incubating the cells in pH-adjusted buffers (pH 5.0, 6.0, 7.0, and 8.0) in the presence of the K+/H+ ionophore nigericin (10 μM) (3). The cytosolic pH was determined by measuring the fluorescence of BCECF (Life Technologies) as described previously (3).

### TIRF microscopy

Cells were transfected with CD63-pHluorin constructs (38) and seeded onto poly-L-lysine–coated FluoroDishes (World Precision Instruments). Imaging was performed using TIRF microscopy. Data analysis was conducted using ImageJ, following the protocols outlined previously (38).

## Data availability

The authors confirm that the data supporting the findings of this study are available within the article and its supporting information.

## Supporting information

This article contains supporting Information.

## Conflict of interest

The authors declare that they have no conflicts of interest with the contents of the article.

## Figures and Tables

**Fig. S3:**