# Supplementary material for: Sodium proton exchanger NHE9 pHine-tunes exosome production by impairing Rab7 activity
Source: J Biol Chem. 2025 Feb 3;301(3):108264. doi: 10.1016/j.jbc.2025.108264 (PMC11929068; doi:10.1016/j.jbc.2025.108264)
Supplement: Supplementary Information [file mmc1.docx]

**Supplementary Information**

**Figure Legends**

**Figure S1. (A)** Upper panel: Representative immunoblot showing NHE9 expression in control Vero E6 cells and NHE9-overexpressing (NHE9+). Lower panel: Graphical representation of the average band intensity from densitometric scans of immunoblots from three biological replicates. Error bars indicate standard deviation (SD). **P < 0.005 ; statistical analysis performed using Student’s t-test. **(B)** Upper panel: Representative immunoblot showing NHE9 expression in control U251 cells and NHE9-overexpressing (NHE9+). Lower panel: Graphical representation of the average band intensity from densitometric scans of immunoblots from three biological replicates. Error bars indicate standard deviation (SD). **P < 0.005 ; statistical analysis performed using Student’s t-test.

**Figure S2. (A)** Graph represents Manders’ Overlap Coefficients (MOC) at 5, 15, and 20 minutes after internalization of dextran tagged to Cascade Blue, comparing colocalization with Rab7 in control and NHE9+ HEK293T cells. Data represent the average of three biological replicates. Error bars indicate standard deviation (SD). NSS: Not statistically significant. Statistical analysis was performed using Student’s t-test. **(B)** Graph represents cytoplasmic pH as determined by measuring the fluorescence emission of BCECF at pH-sensitive and pH-insensitive wavelengths, calibrated in buffers of known pH. Data represent the average of three biological replicates. Error bars indicate standard deviation (SD). NSS: Not statistically significant. Statistical analysis was performed using Student’s t-test.

**Figure S3**. Top panel: Immunoblot showing Rab7-GTP expression in control HEK293T cells and NHE9+ cells expressing the constitutively active mutant Rab7^Q67L. Bottom panel: Graphical representation of the average band intensity from densitometric scans of immunoblots showing Rab7 expression in control HEK293T cells and NHE9+ cells expressing the constitutively active mutant Rab7^Q67L from three biological replicates. Error bars indicate standard deviation (SD). NSS: Not statistically significant. Statistical analysis was performed using Student’s t-test.

**Video 1:** Time lapse imaging of CD63-pHluorin in control HEK293-T cells.

**Video 2:** Time lapse imaging of CD63-pHluorin in NHE9+ HEK293-T cells.
